# Supplementary material for: CXCL1/CXCR2 Paracrine Axis Contributes to Lung Metastasis in Osteosarcoma
Source: Cancers (Basel). 2020 Feb 17;12(2):459. doi: 10.3390/cancers12020459 (PMC7072404; doi:10.3390/cancers12020459)
Supplement: Supplementary file 1 [file cancers-12-00459-s001.pdf]

Figure 1B

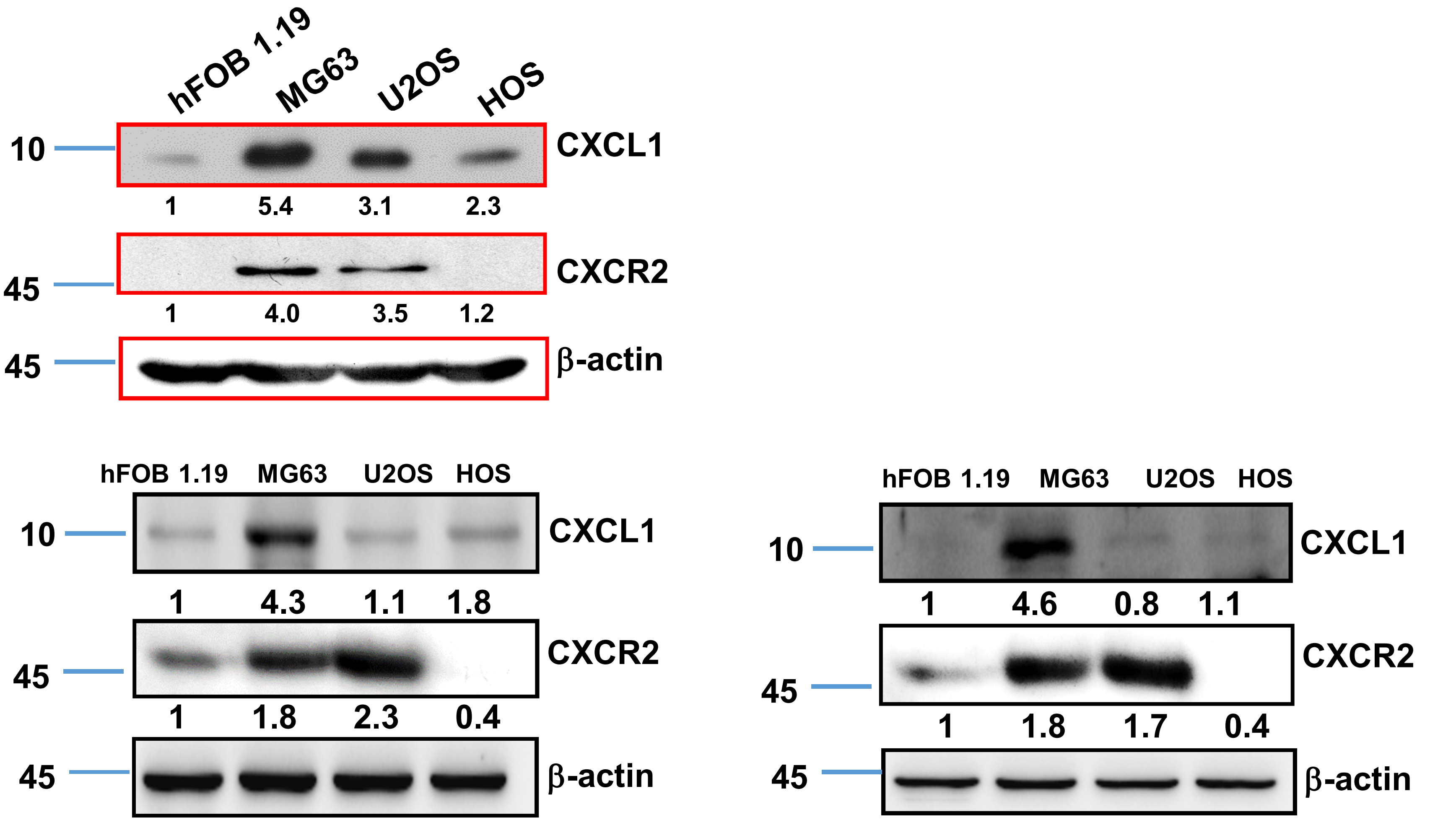
hFOB 1.19
MG63
U2OS
HOS
10
CXCL1
1
5.4
3.1
2.3
45
CXCR2
1
4.0
3.5
1.2
45
β-actin
hFOB 1.19 MG63 U2OS HOS
10
CXCL1
1 4.3 1.1 1.8
45
CXCR2
1 1.8 2.3 0.4
45
β-actin
hFOB 1.19 MG63 U2OS HOS
10
CXCL1
1 4.6 0.8 1.1
45
CXCR2
1 1.8 1.7 0.4
45
β-actin

Figure 4B

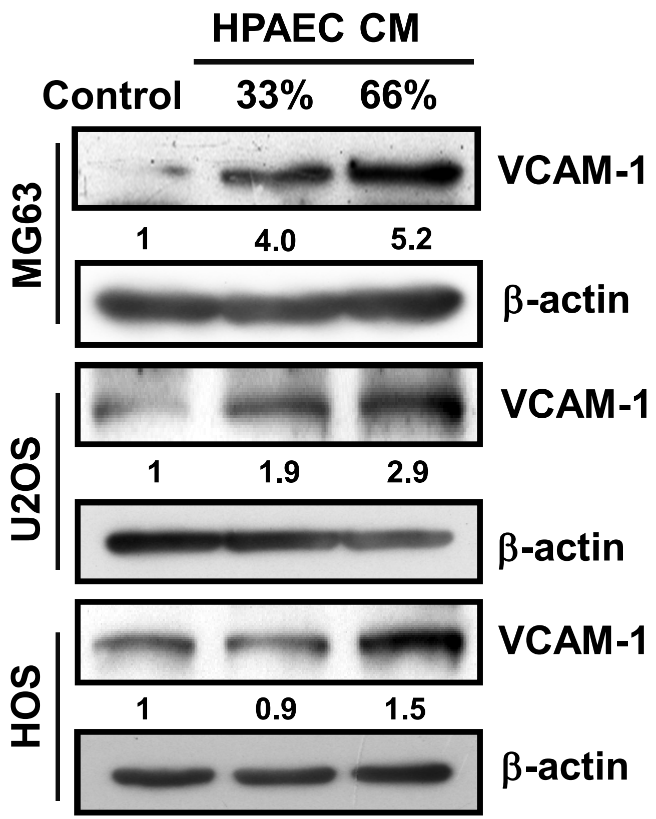

HPAEC CM
Control
33%
66%
MG63
VCAM-1
1
4.0
5.2
β-actin
U2OS
VCAM-1
1
1.9
2.9
β-actin
HOS
VCAM-1
1
0.9
1.5
β-actin

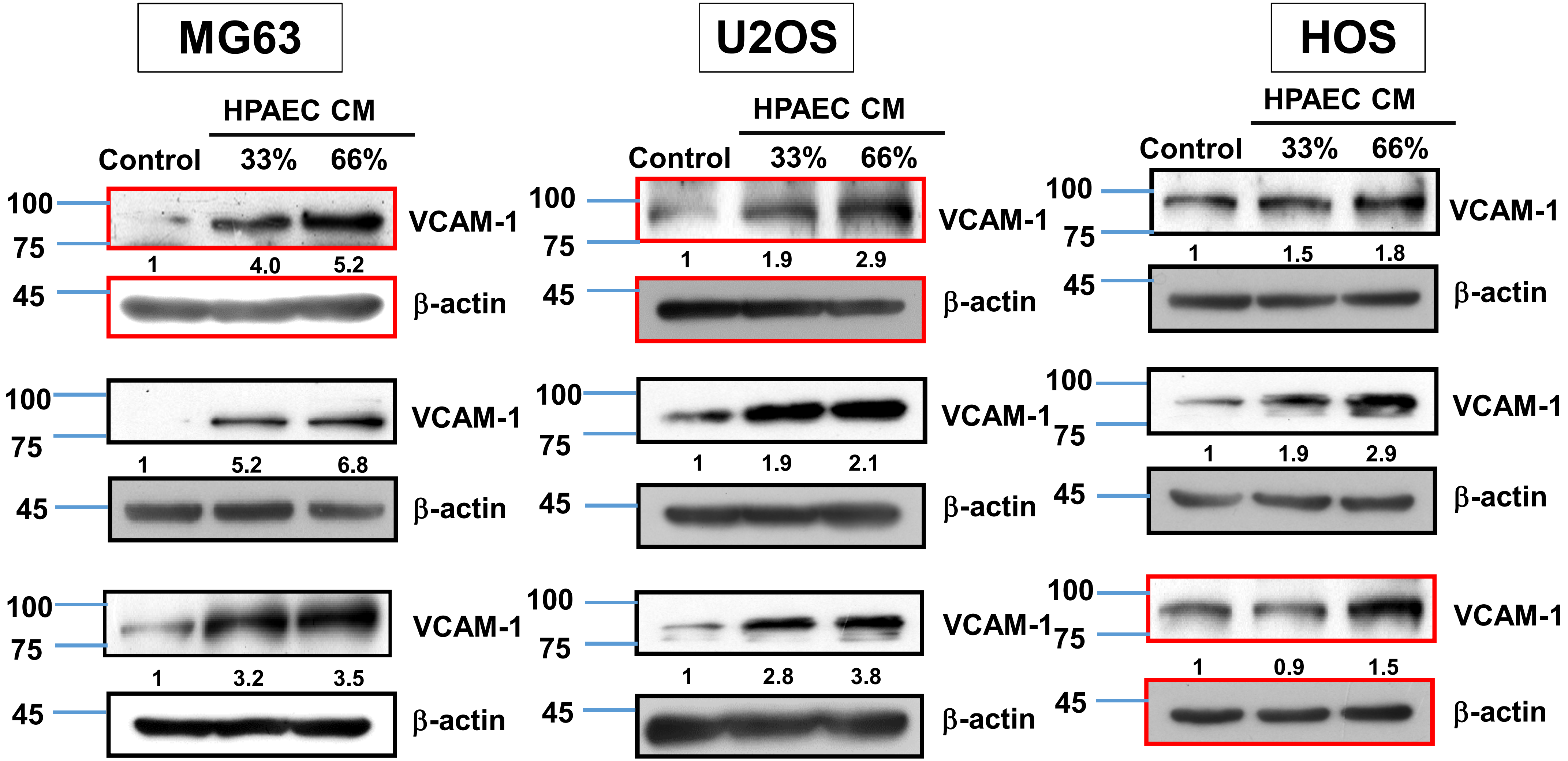
MG63
U2OS
HOS
HPAEC CM
Control
33%
66%
100
75
45
VCAM-1
β-actin
1
4.0
5.2
5.2
6.8
3.2
3.5
1.9
2.9
2.1
2.8
3.8
1.5
1.8
0.9

Figure 4D

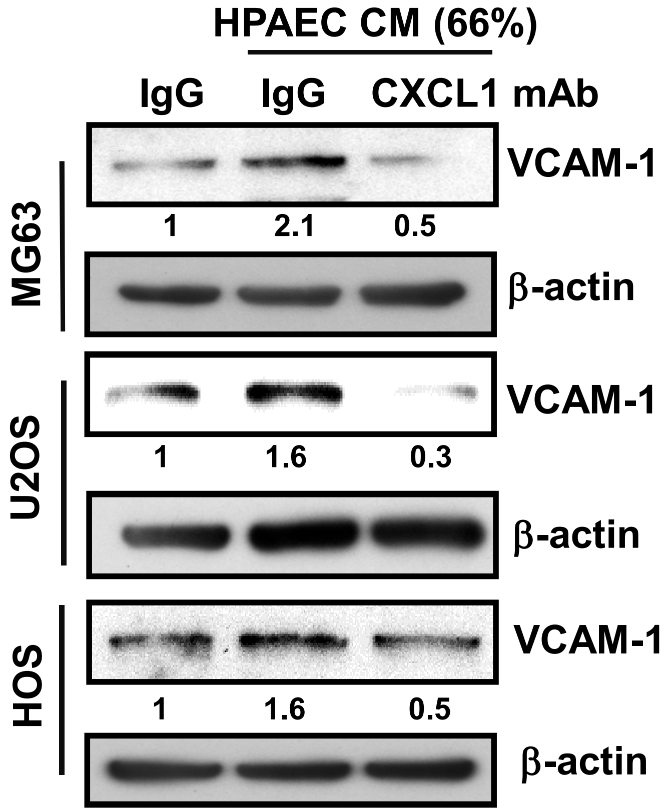

HPAEC CM (66%)
IgG
IgG
CXCL1 mAb
MG63
VCAM-1
1
2.1
0.5
β-actin
U2OS
VCAM-1
1
1.6
0.3
β-actin
HOS
VCAM-1
1
1.6
0.5
β-actin

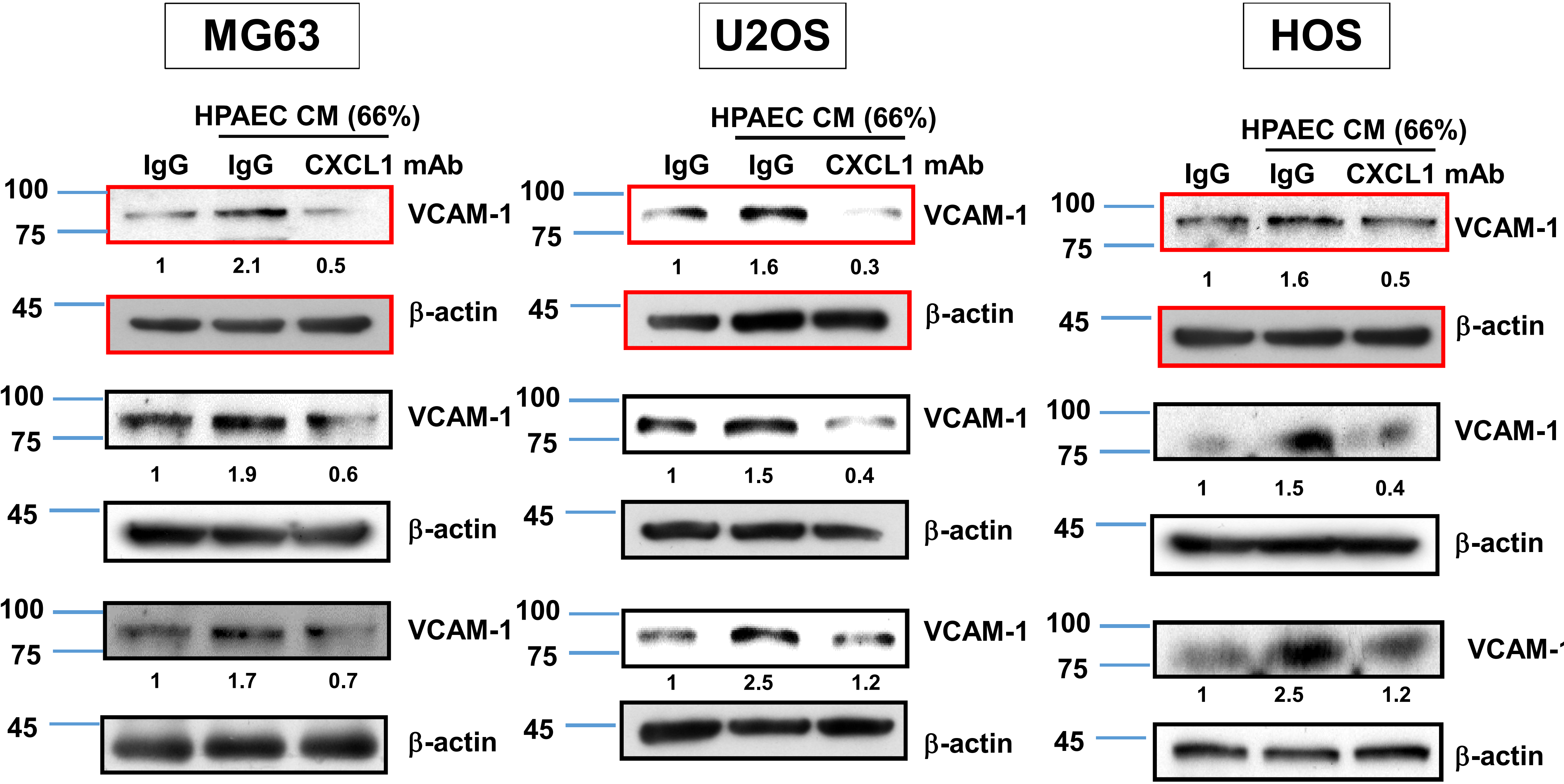
MG63
U2OS
HOS
HPAEC CM (66%)
IgG IgG CXCL1 mAb
100
75
VCAM-1
1 2.1 0.5
45
β-actin
1 1.9 0.6
1 1.7 0.7
1 1.6 0.3
1 1.5 0.4
1 2.5 1.2
1 1.6 0.5

Figure 4F

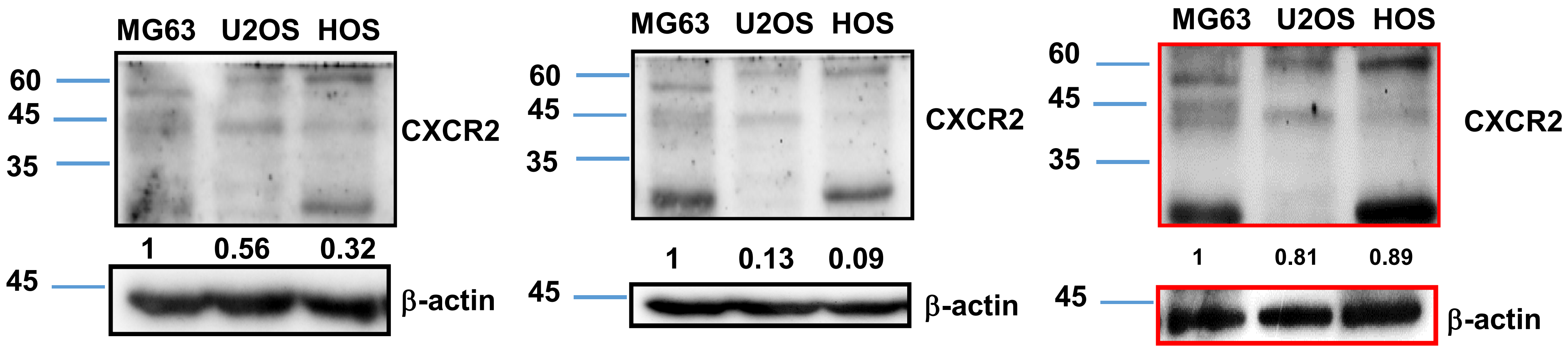
MG63 U2OS HOS
60
45
35
CXCR2
1 0.56 0.32
45
β-actin
MG63 U2OS HOS
60
45
35
CXCR2
1 0.13 0.09
45
β-actin
MG63 U2OS HOS
60
45
35
CXCR2
1 0.81 0.89
45
β-actin

Figure 4J

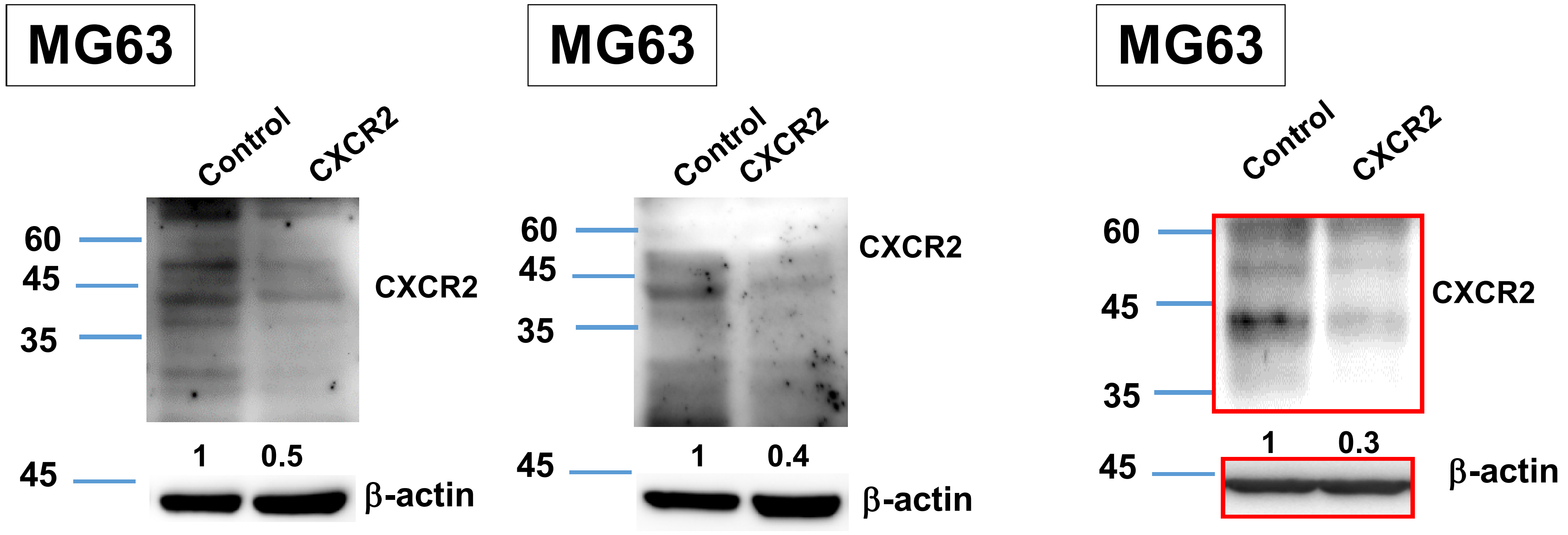
MG63
Control
CXCR2
60
45
35
CXCR2
1
0.5
45
β-actin
MG63
Control
CXCR2
60
45
35
CXCR2
1
0.4
45
β-actin
MG63
Control
CXCR2
60
45
35
CXCR2
1
0.3
45
β-actin

Figure 5C

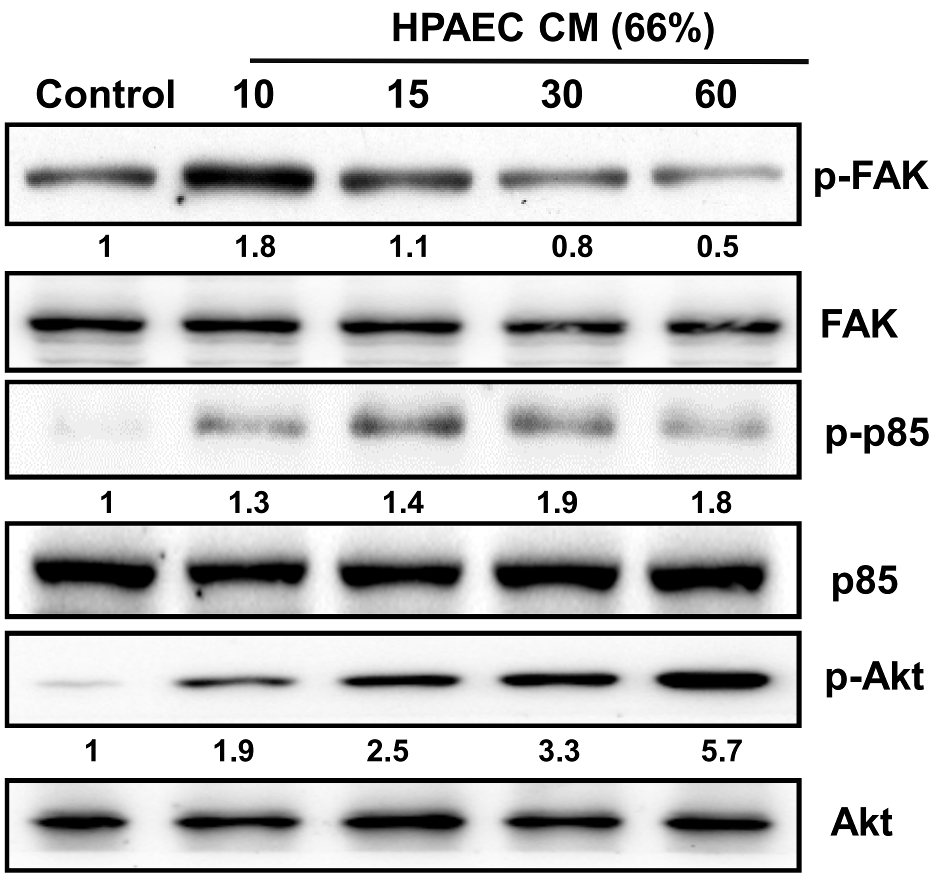

HPAEC CM (66%)
Control
10
15
30
60
p-FAK
1
1.8
1.1
0.8
0.5
FAK
p-p85
1
1.3
1.4
1.9
1.8
p85
p-Akt
1
1.9
2.5
3.3
5.7
Akt

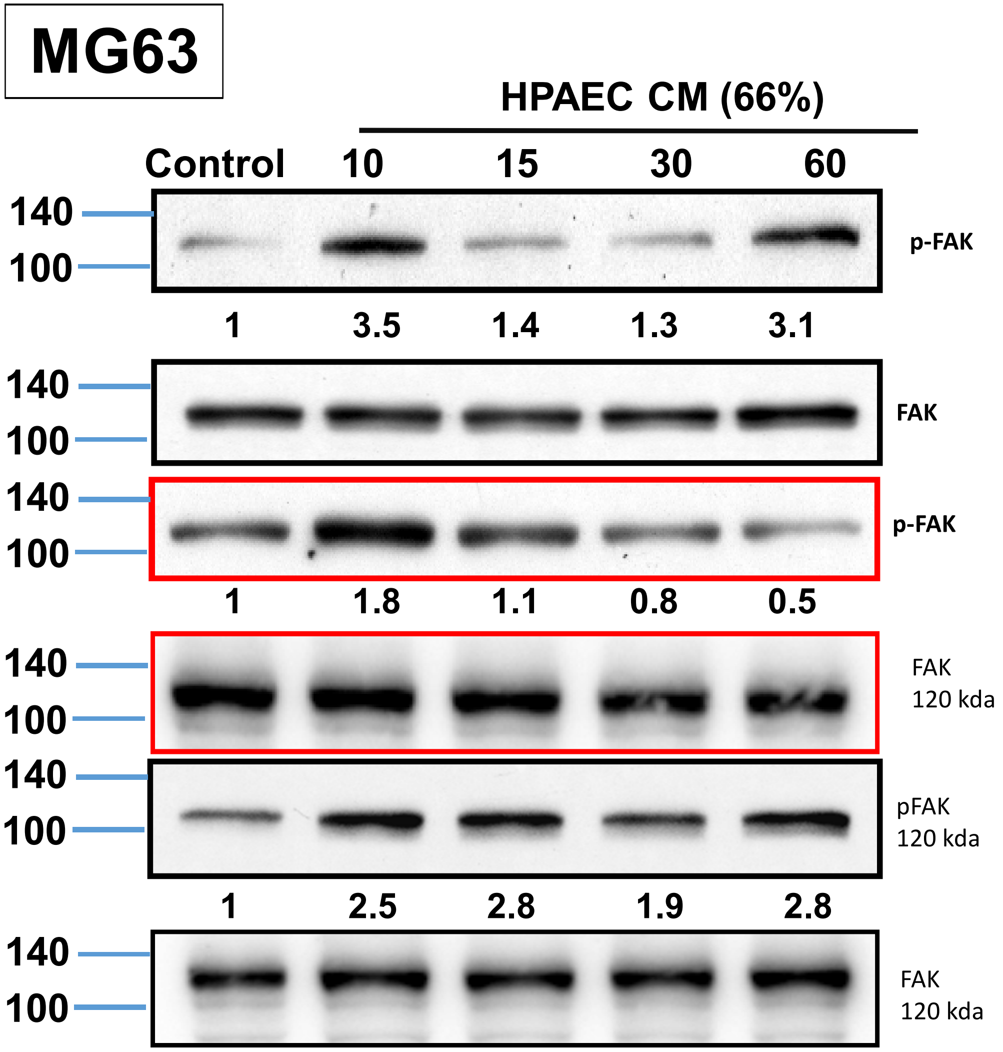
MG63
HPAEC CM (66%)
Control
10
15
30
60
140
100
p-FAK
1
3.5
1.4
1.3
3.1
FAK
p-FAK
1
1.8
1.1
0.8
0.5
FAK
120 kda
pFAK
120 kda
1
2.5
2.8
1.9
2.8
FAK
120 kda

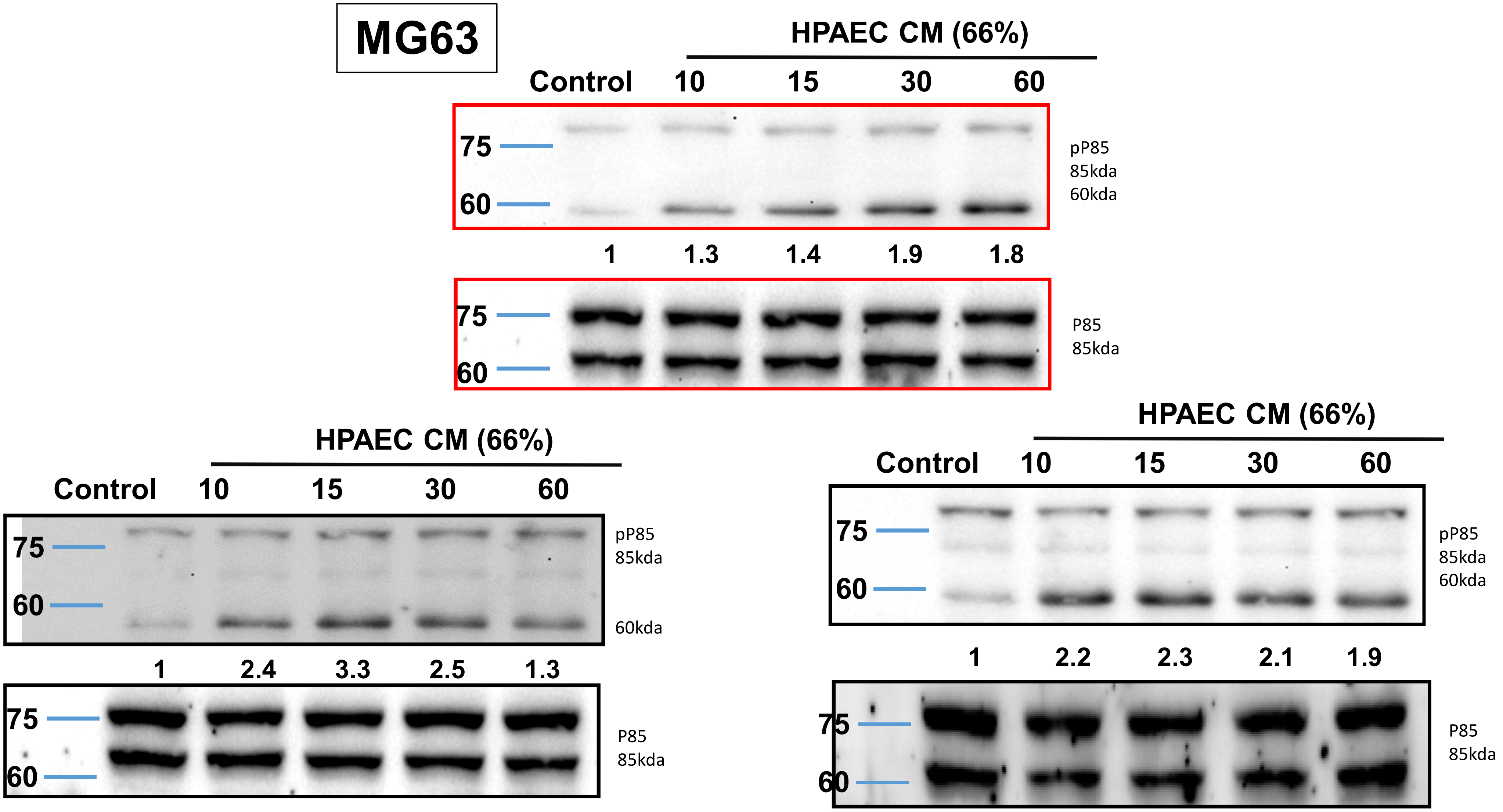
MG63
HPAEC CM (66%)
Control
10
15
30
60
75
60
pP85
85kda
60kda
1
1.3
1.4
1.9
1.8
75
60
P85
85kda
HPAEC CM (66%)
Control
10
15
30
60
75
60
pP85
85kda
60kda
1
2.4
3.3
2.5
1.3
75
60
P85
85kda
HPAEC CM (66%)
Control
10
15
30
60
75
60
pP85
85kda
60kda
1
2.2
2.3
2.1
1.9
75
60
P85
85kda

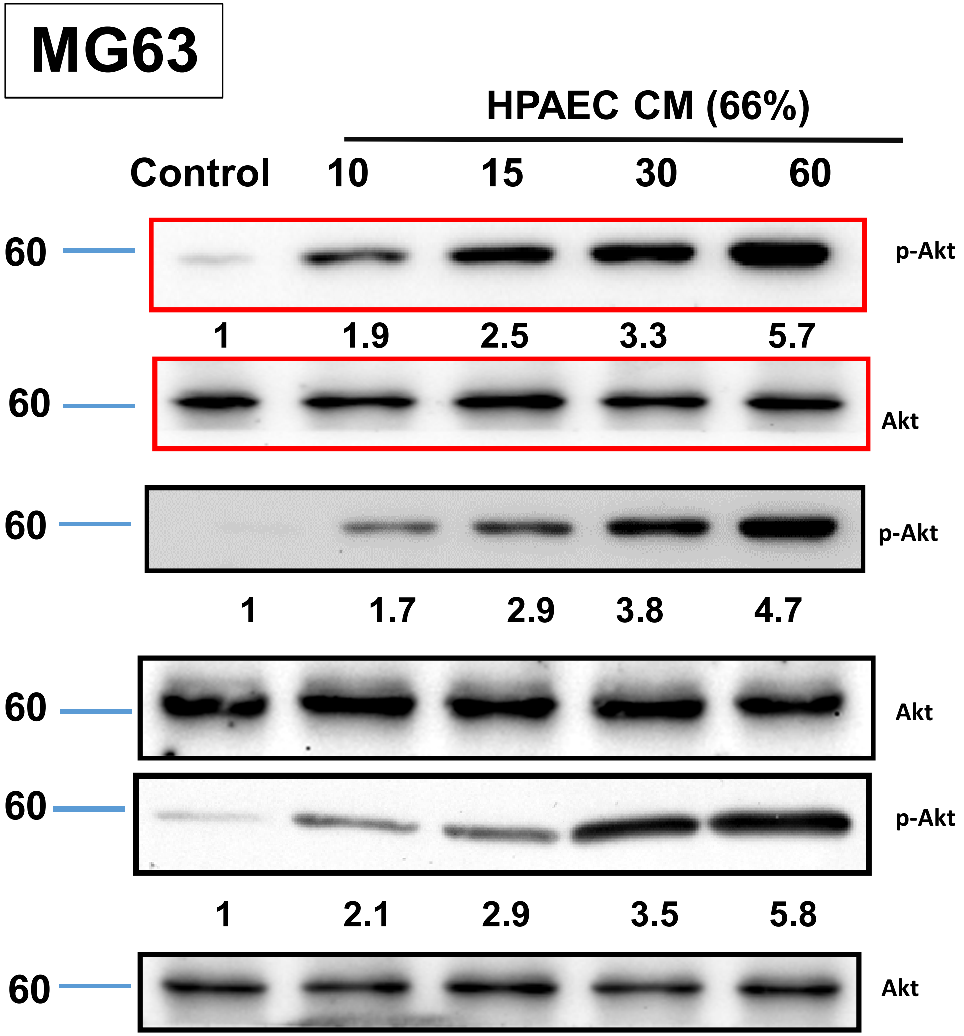

MG63
HPAEC CM (66%)
Control
10
15
30
60
60
p-Akt
1
1.9
2.5
3.3
5.7
60
Akt
60
p-Akt
1
1.7
2.9
3.8
4.7
60
Akt
60
p-Akt
1
2.1
2.9
3.5
5.8
60
Akt

Figure 5D

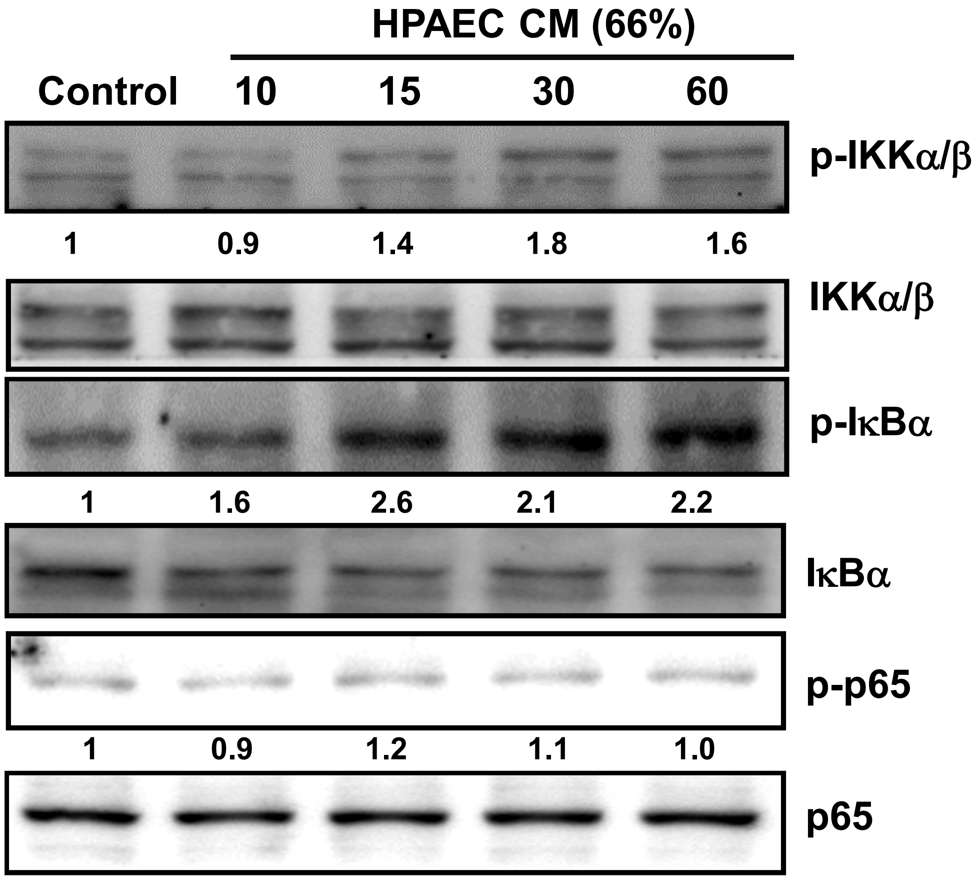
HPAEC CM (66%)
Control
10
15
30
60
p-IKKα/β
1
0.9
1.4
1.8
1.6
IKKα/β
p-IκBα
1
1.6
2.6
2.1
2.2
IκBα
p-p65
1
0.9
1.2
1.1
1.0
p65

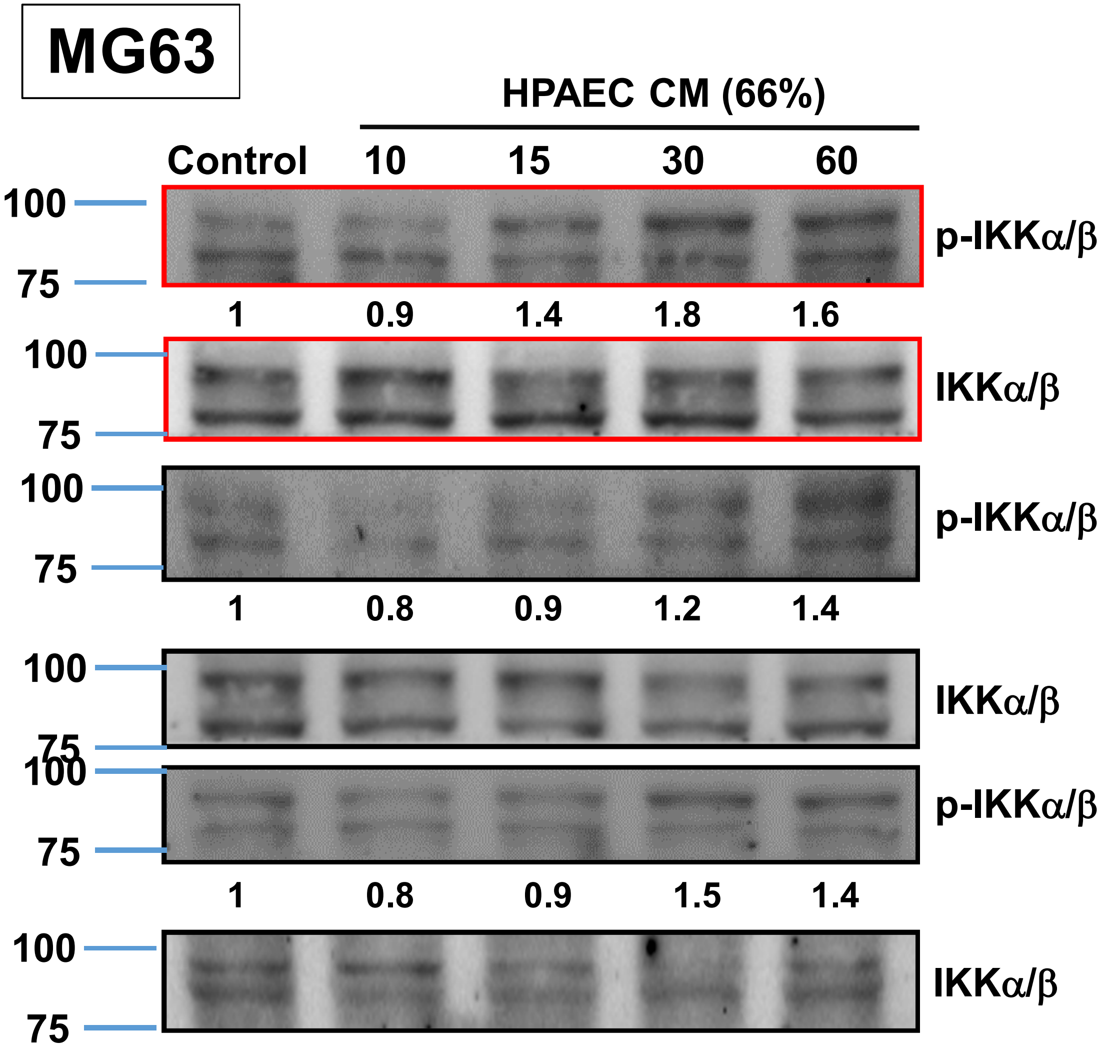
MG63
HPAEC CM (66%)
Control
10
15
30
60
100
75
p-IKKα/β
1
0.9
1.4
1.8
1.6
IKKα/β
p-IKKα/β
1
0.8
0.9
1.2
1.4
IKKα/β
p-IKKα/β
1
0.8
0.9
1.5
1.4
IKKα/β

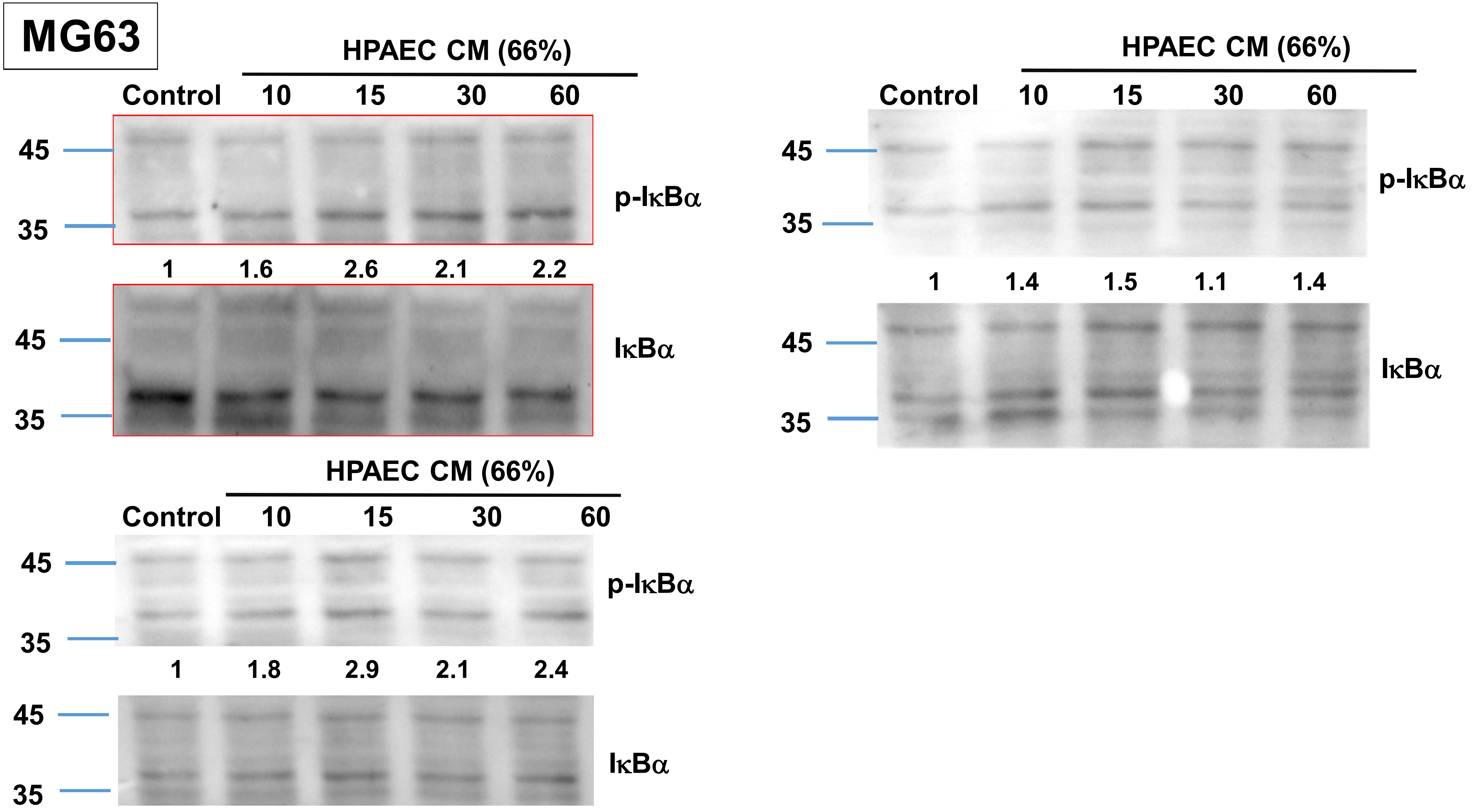
MG63
HPAEC CM (66%)
Control 10 15 30 60
45
35
p-IκBα
1 1.6 2.6 2.1 2.2
45
35
IκBα
HPAEC CM (66%)
Control 10 15 30 60
45
35
p-IκBα
1 1.8 2.9 2.1 2.4
45
35
IκBα
HPAEC CM (66%)
Control 10 15 30 60
45
35
p-IκBα
1 1.4 1.5 1.1 1.4
45
35
IκBα

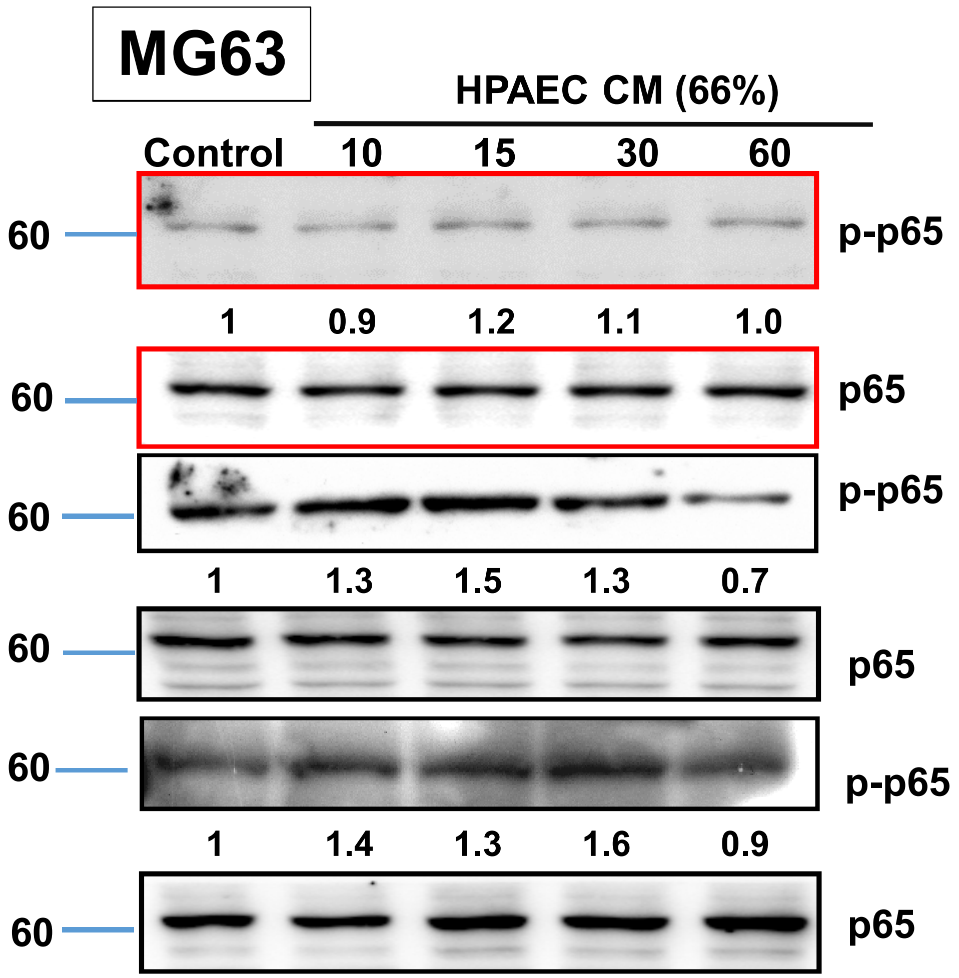
MG63
HPAEC CM (66%)
Control
10
15
30
60
60
p-p65
1
0.9
1.2
1.1
1.0
60
p65
60
p-p65
1
1.3
1.5
1.3
0.7
60
p65
60
p-p65
1
1.4
1.3
1.6
0.9
60
p65

Fig 6A

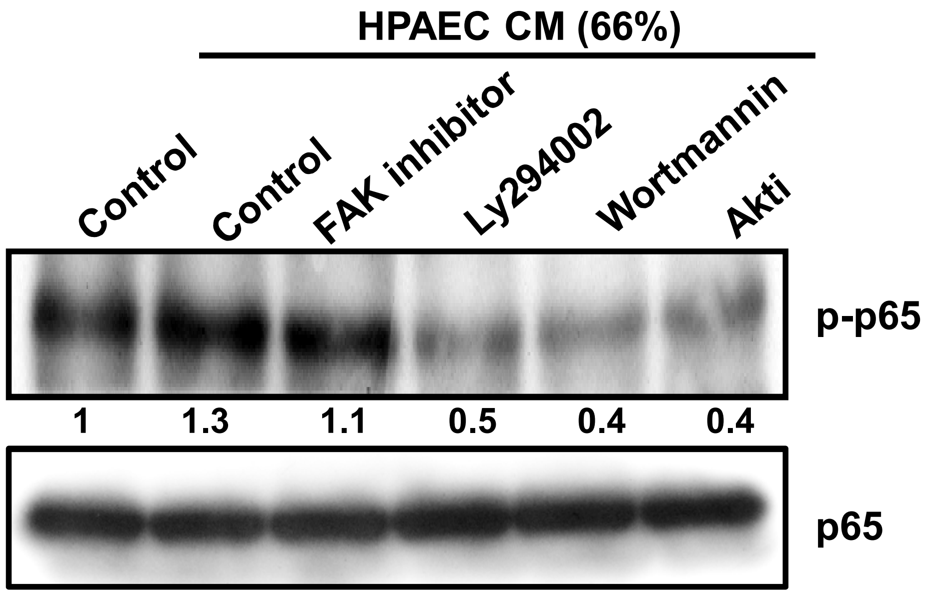
HPAEC CM (66%)
Control
Control
FAK inhibitor
Ly294002
Wortmannin
Akti
p-p65
1
1.3
1.1
0.5
0.4
0.4
p65

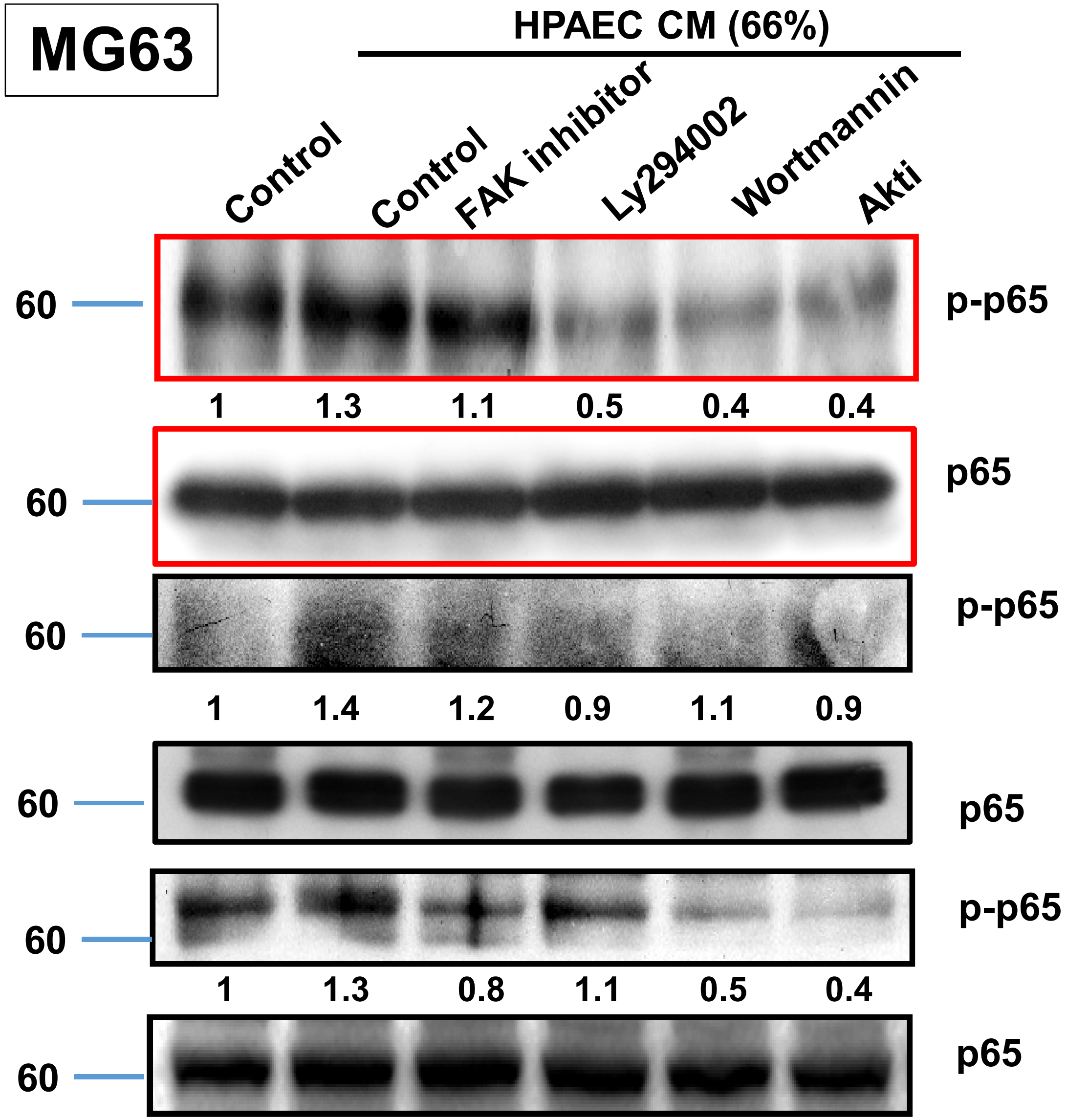
MG63
HPAEC CM (66%)
Control
Control
FAK inhibitor
Ly294002
Wortmannin
Akti
60
p-p65
1
1.3
1.1
0.5
0.4
0.4
60
p65
60
p-p65
1
1.4
1.2
0.9
1.1
0.9
60
p65
60
p-p65
1
1.3
0.8
1.1
0.5
0.4
60
p65
